# Helical Polyacetylenes Induced via Noncovalent Chiral Interactions and Their Applications as Chiral Materials

**DOI:** 10.1007/s41061-017-0161-4

**Published:** 2017-07-20

**Authors:** Katsuhiro Maeda, Eiji Yashima

**Affiliations:** 1 Graduate School of Natural Science and Technology, Kanazawa University, Kakuma-machi, Kanazawa, 920-1192 Japan; 2 Department of Molecular Design and Engineering, Graduate School of Engineering, Nagoya University, Chikusa-ku, Nagoya, 464-8603 Japan

**Keywords:** Polyacetylene, Helix, Helicity induction, Noncovalent chiral interaction, Memory, Chiral amplification, Chiral recognition ability, Chiral stationary phase

## Abstract

Construction of predominantly one-handed helical polyacetylenes with a desired helix sense utilizing noncovalent chiral interactions with nonracemic chiral guest compounds based on a supramolecular approach is described. As with the conventional dynamic helical polymers possessing optically active pendant groups covalently bonded to the polymer chains, this noncovalent helicity induction system can show significant chiral amplification phenomena, in which the chiral information of the nonracemic guests can transfer with high cooperativity through noncovalent bonding interactions to induce an almost single-handed helical conformation in the polymer backbone. An intriguing “memory effect” of the induced macromolecular helicity is observed for some polyacetylenes, which means that the helical conformations induced in dynamic helical polyacetylene can be transformed into metastable static ones by tuning their helix-inversion barriers. Potential applications of helical polyacetylenes with controlled helix sense constructed by the “noncovalent helicity induction and/or memory effect” as chiral materials are also described.

## Introduction

The helix is a chiral topological structure; therefore, a polymer chain can become optically active despite the absence of any asymmetric carbons and stereogenic centers when it predominantly folds into either a left- or right-handed helical conformation. The development of synthetic helical polymers with a controlled helicity has drawn considerable attention in the fields of polymer and supramolecular chemistry because of their wide variety of potential applications in materials science, such as chiral adsorbents for separating enantiomers, catalysis for asymmetric reactions, ferroelectric liquid crystals (LCs), and nonlinear optical materials [1–13]. Until now, many helical polymers have been synthesized, and these polymers can be mainly classified as either static helical polymers or dynamic helical polymers from the perspective of helix inversion barriers [1, 4, 7]. Static helical polymers are characterized by helix inversion barriers significantly high enough to maintain their helical conformations even in solution. This class of helical polymers usually possesses bulky pendant groups as represented by poly(triphenylmethyl methacrylate) (PTrMA), and the large steric repulsion between these bulky pendants contributes to their high helix inversion barriers [11]. Therefore, static helical polymers with a one-handed helical conformation can be synthesized by the helix-sense-selective polymerization of the corresponding monomers using chiral initiators or catalysts, where the predominant helix sense is determined during the polymerization under kinetic control. On the other hand, dynamic helical polymers are characterized by a relatively low helix inversion barrier, which enables the polymer backbone to interconvert rapidly between the right- and left-handed helical conformations in a single polymer chain in solution. However, the helix reversal states separating right- and left-handed helical segments are disadvantageous in energy, so helix reversals occur infrequently in polymer chains, meaning that dynamic helical polymers tend to have a long helical persistence length. Therefore, optically active polymers with a large excess of single-handed helical conformation can be obtained by the copolymerization of achiral monomers with a small amount of optically active ones or the copolymerization of nonracemic monomers with a small enantiomeric excess (ee) [12, 13]. These two chiral amplification phenomena, which were termed the “sergeants and soldiers” effect [14] and the “majority rule” principle [15], respectively, are the most intriguing features of dynamic helical polymers.

Substituted polyacetylenes, which can be synthesized by the polymerization of the corresponding acetylene monomers, are one of the well-studied π-conjugated polymers and usually belong to a class of dynamic helical polymers because of their low helix inversion barriers [1, 4]. Prior to the groundbreaking discovery of the metallic conductivity in doped polyacetylene, Ciardelli et al. synthesized mono-substituted polyacetylenes bearing optically active pendants and pointed out that these polymers formed a predominantly one-handed helical conformation because they showed an intense induced circular dichroism (ICD) in the absorption region because of the conjugated polyene backbone [16]. Since this pioneering work, a wide variety of predominantly one-handed helical polyacetylenes have been synthesized by the polymerization of the corresponding optically active monomers owing to the development of transition metal-based catalysts effective for the polymerization of acetylene monomers [3, 17–20]. Especially rhodium catalysts enabled the synthesis of high-molecular-weight stereoregular polyacetylenes bearing various functional pendants by directly polymerizing the corresponding acetylene monomers because of their high tolerance toward polar functional groups [21–25]. Moreover, it has been revealed that the control of stereoregularity in the main-chain structure is essential for the formation of a helical conformation in polyacetylenes [21, 26, 27]. Taking advantage of chiral amplification phenomena in dynamic helical polymers, predominantly one-handed helical polyacetylenes have also been prepared by the copolymerization of achiral acetylene monomers with a small amount of optically active ones.

In this chapter, we focus on the synthesis of helical polyacetylenes with controlled helix sense by noncovalent macromolecular helicity induction through interactions with chiral compounds. Some examples of potential applications of helical polyacetylenes constructed by noncovalent helicity induction to chiral separation, asymmetric synthesis, and the template for organizing functional molecules in a helical array are also described.

## Macromolecular Helicity Induction in Polyacetylenes

**Fig. 1:**
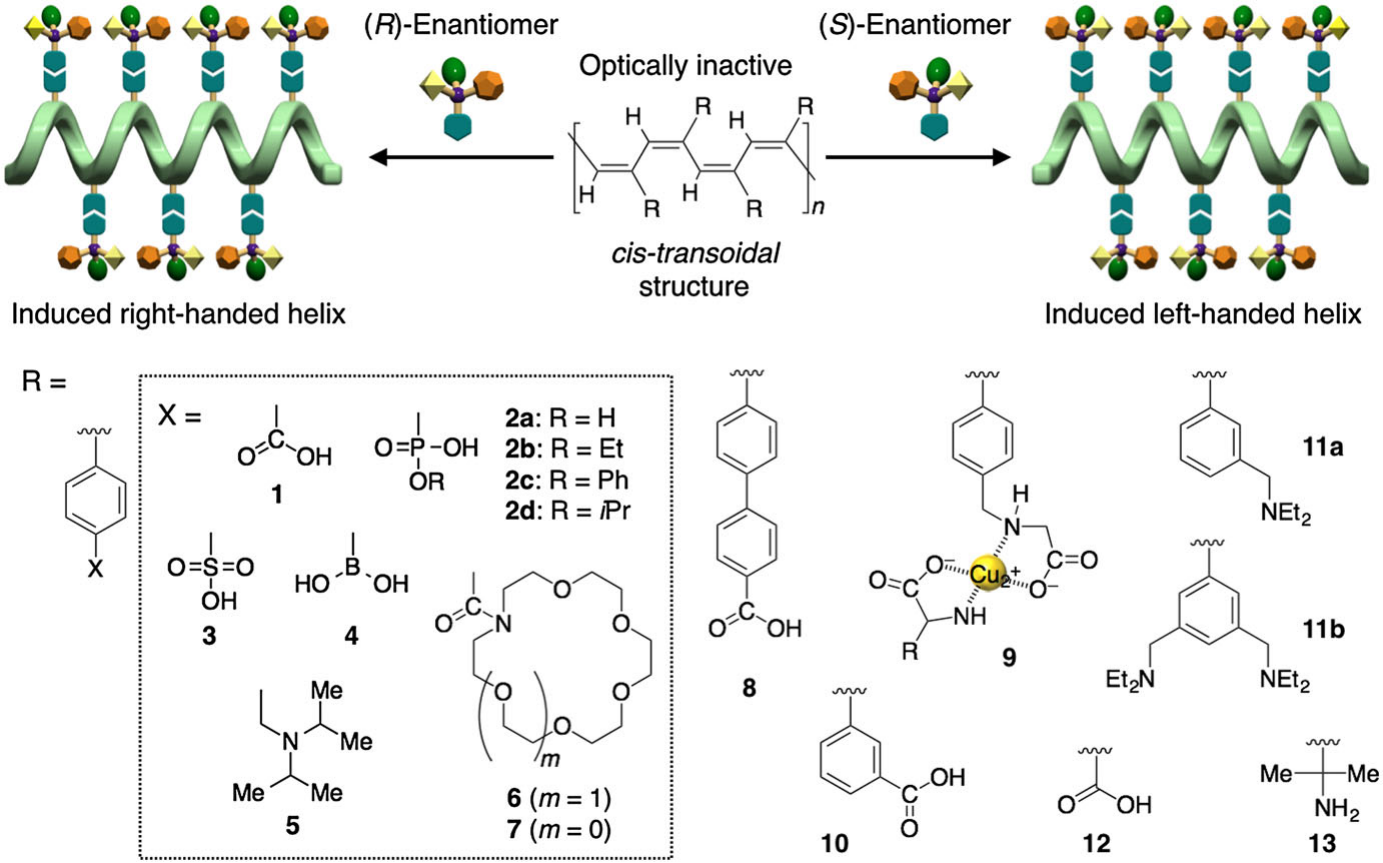
Schematic illustration of preferred-handed macromolecular helicity induction in polyacetylenes (**1**–**13**) via noncovalent interaction with optically active compounds

**Fig. 2:**
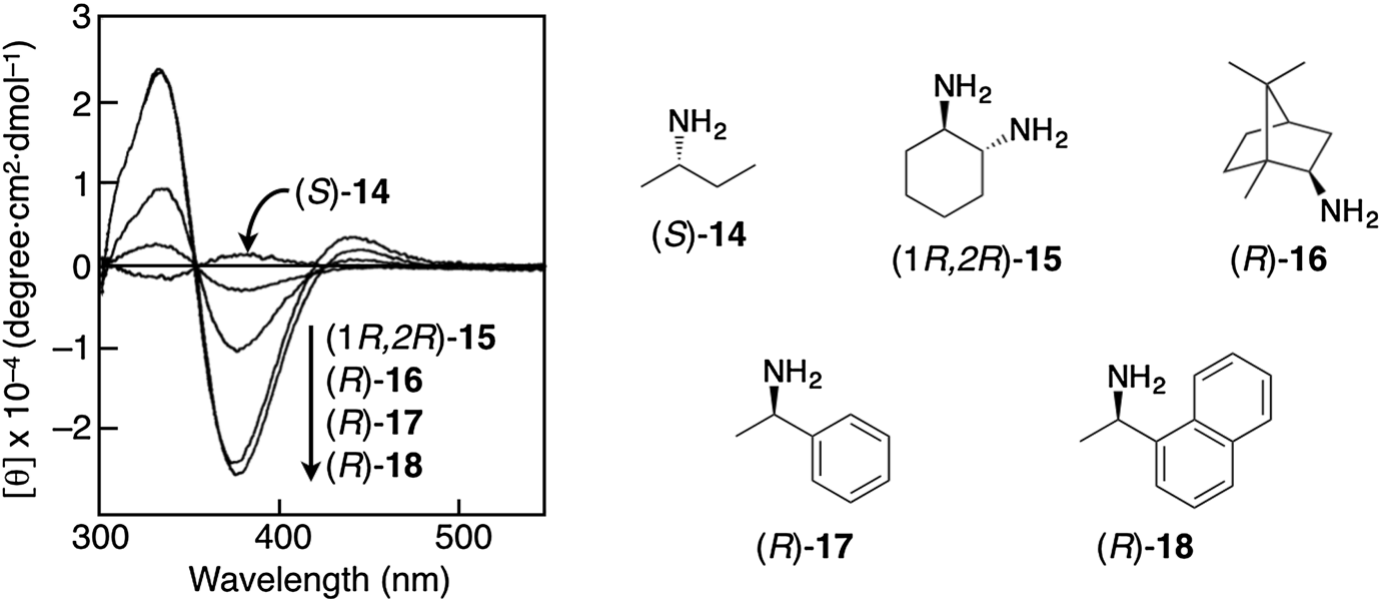
CD spectra of **1** with chiral amines (**14**–**18**) in DMSO (Reprinted with permission from [29]. Copyright 1997 American Chemical Society)

A predominantly one-handed helical conformation can be induced in optically inactive polyacetylenes through noncovalent bonding interaction with optically active compounds (Fig. 1). A stereoregular (*cis*-*transoidal*) poly(phenylacetylene)-bearing carboxy pendant (**1**) is the first example of such a helical polyacetylene, whose macromolecular helicity with an excess helical sense is induced by noncovalent chiral acid-base interactions [28]. When **1** is complexed with optically active amines such as **14**–**18** in DMSO, a preferred-handed helical conformation can be immediately induced in the polymer backbone of **1** to exhibit a characteristic ICD in the long absorption region because of the conjugated double bonds of the polymer backbone (Fig. 2). Since the same Cotton effect signs were observed for the complexes of **1** with various primary amines and amino alcohols when their absolute configurations are the same, the sign of the Cotton effect induced in **1** allows us to assign the configurations of primary chiral amines [29]. Taking advantage of this noncovalent macromolecular helicity induction strategy, the predominant helix sense can be induced in dynamic helical polyacetylenes in a controlled manner upon complexation with chiral guests after polymerization. Therefore, a series of polyacetylenes capable of forming a preferred-handed helical conformation in response to the chirality of target chiral compounds has been designed and synthesized by introducing a specific functional group into the pendants (**2**–**13**) (Fig. 1) [30–52].

**Fig. 3:**
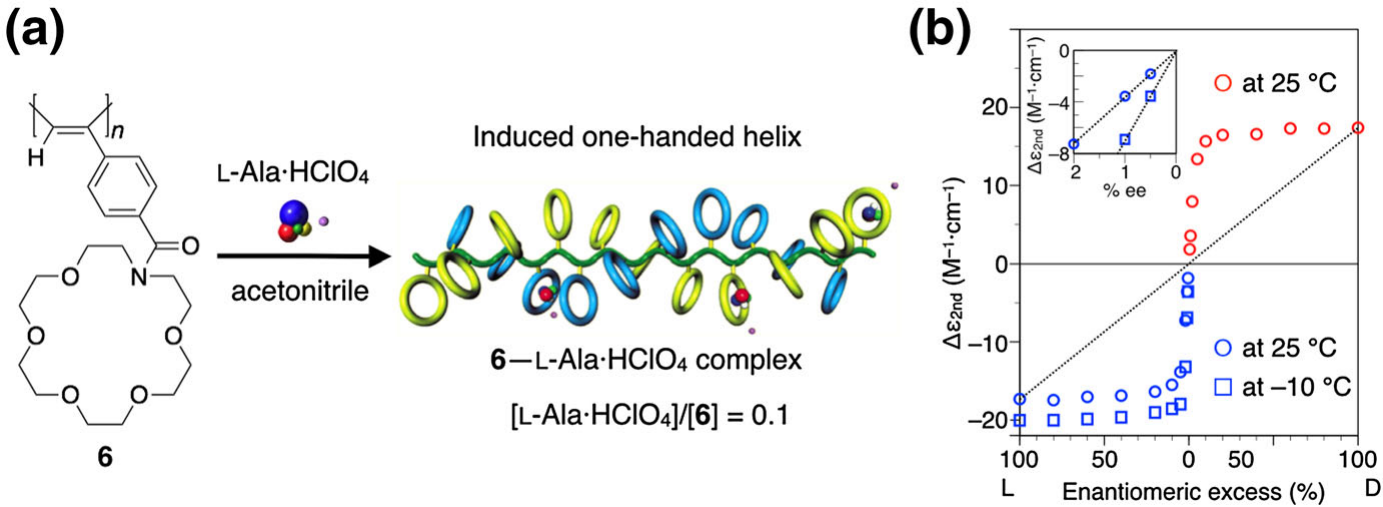
Schematic illustration of a helix induction with an excess handedness in **6** upon complexation with a small amount of l-Ala·HClO_4_ in acetonitrile (**a**). Plots of ICD intensity changes of **6** against the % ee of Ala·HClO_4_ (**b**) (Reprinted with permission from [39]. Copyright 2003 American Chemical Society)

Two quintessential chiral amplification phenomena, that is, the “sergeants and soldiers” effect and “majority rule,” are also observed in dynamic helical polyacetylenes during the noncovalent helicity induction process. Especially a poly(phenylacetylene) possessing an aza-18-crown-6-ether residue as the functional pendant (**6**) showed the highest degree of chiral amplification through a noncovalent interaction with chiral amino acids (Fig. 3) [39]. For example, **6** formed an almost one-handed helix in the presence of 0.1 equiv. of l-alanine perchlorate (l-Ala·HClO_4_) in acetonitrile and exhibited a distinct ICD even in the presence of a tiny amount of l-Ala·HClO_4_ ([l-Ala·HClO_4_]/[**6**] = 0.01), which can be regarded as a representative example of the “sergeants and soldiers” effect in the noncovalent helicity induction system (Fig. 3a). More interestingly, **6** showed the almost full ICD in response to the chirality of nonracemic Ala·HClO_4_ with a very low ee of 5% (Fig. 3b). This prominent majority rule effect of **6** enables unambiguous detection of an extremely small optical activity of the amino acids by measuring CD. For instance, **6** showed a detectable ICD with accuracy and reproducibility even in the presence of nearly racemic Ala with as low as 0.005% ee [39]. This remarkably high sensitivity to the chirality of chiral molecules observed for **6** is attributable to the rigid main-chain structure possessing the bulky substituents, which may significantly increase the helical persistence length separated by infrequently occurring helix reversals in **6**. Moreover, **6** exhibited the same Cotton effect signs in response to the chirality of all the common 19 l-amino acids. Therefore, **6** can be used as a reliable and practically useful synthetic receptor for the rapid detection and precise determination of small enantiomeric imbalances in chiral natural and non-natural amino acids [39, 53, 54].

**Fig. 4:**
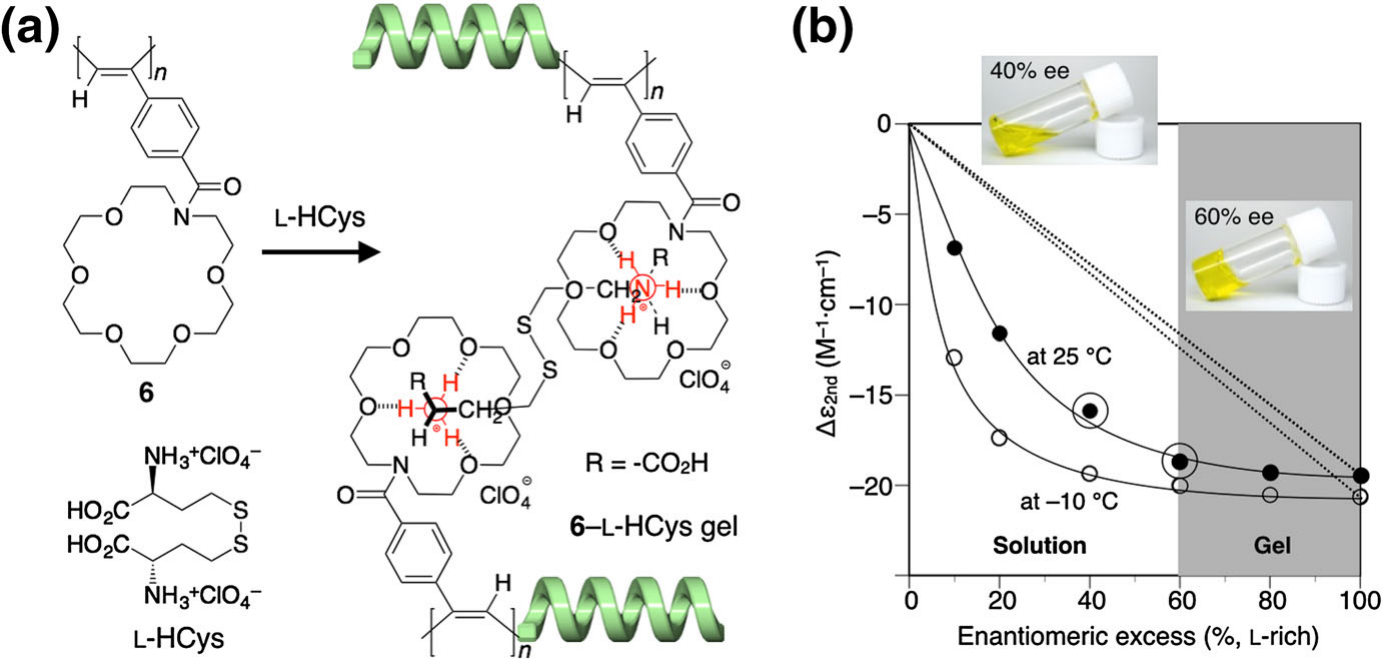
Schematic illustration of a helicity induction with an excess handedness in **6** with l-HCys and the gelation by intermolecular cross-linking through noncovalent bonding between the pendant crown ether units and the two ammonium groups of l-HCys (**a**). Plots of ICD intensity changes of **6** against the % ee of HCys (l rich) in acetonitrile. *Inset* shows the photographs of **6** complexed with nonracemic HCys (40 and 60% ee) (**b**) (Reprinted with permission from [55]. Copyright 2005 American Chemical Society)

The polyacetylene **6** also folded into a predominantly one-handed helical conformation with nonracemic bis(amino acid)s such as l-homocystine perchlorate (l-HCys·HClO_4_) in acetonitrile, thus producing an organogel by noncovalent intermolecular cross-linking between the aza-18-crown-6 ether pendants and the bis(amino acid)s (Fig. 4) [55]. Interestingly, **6** formed an organogel only when the ee of HCys was higher than 60% ee, at which the complex showed a full ICD as intense as that in the presence of optically pure HCys, while no gelation took place in the presence of racemic HCys and analogous achiral diamines. Therefore, the gelation of **6** is quite sensitive to the chirality of the bis(amino acid)s, and the formation of an almost one-handed helical conformation induced in **6** seems to be essential for the gelation. This is the first example of chirality-responsive gelation of polymers regulated by noncovalent chiral interactions through which one of helices is induced in the polymers to form a gel.

In the case of water-soluble polyacetylenes including the sodium salts of **1** (**1**·Na) and **12** (**12**·Na), the hydrochloride of **5** (**5**·HCl), **2**–**4**, **6**, and **7**, macromolecular helicity induction through noncovalent interaction with chiral compounds is possible even in water [34, 36, 38, 40, 41, 45, 47, 48, 56–58]. For example, the negatively charged **2b** exhibited characteristic ICDs in the polymer backbone regions because of the formation of a predominantly one-handed helical conformation in response to the chirality of a variety of water-soluble biomolecules including amino acids, amino sugars, carboxylic and phosphoric acids, carbohydrates, and peptides in water [45, 48]. As for amino acids, **2b** exhibited clear ICDs in water in response to the chirality of all the common 19 l-amino acids without derivatization, suggesting that this polymer can be used as a powerful chirality-sensing probe for free chiral amino acids in water [45]. Moreover, water-soluble *cis*-*transoidal* polyacetylenes bearing acidic functional pendant groups such as **1**, **2b**, **3**, and **12** can be directly prepared by the stereospecific polymerization of the corresponding acetylene monomers in water using water-soluble rhodium complexes as the catalysts in the presence of bases such as sodium hydroxide or amines [34, 36]. Therefore, both the synthesis of dynamic helical polymers and subsequent preferred-handed helicity induction on the polymers are entirely possible in water without the use of any surfactants and organic solvents.

The positively charged water-soluble polyacetylene bearing a bulky ammonium group as the pendants (**5**·HCl) formed a predominantly one-handed helical conformation in response to the chirality of various chiral acids such as carboxylic, phosphoric, and sulfonic acids in water [47, 59]. The sensitivity of **5**·HCl was extremely high, and an almost single-handed helix was induced in **5**·HCl in the presence of a small amount of chiral acids ([chiral acid]/[**5**·HCl] = 0.1) even with a low ee in water. On the other hand, the neutral **5** is not as sensitive as **5**·HCl in organic solvents, and a large amount of chiral acids is required to show the full ICD. Therefore, the polyelectrolyte function of **5**·HCl plays an important role in such a high chiral amplification in water. The thermodynamic parameters featuring the dynamic helical structures were estimated for optically active copolymers consisting of achiral phenylacetylene units bearing charged pendant groups and a small amount of a neutral chiral phenylacetylene unit in water and organic solvents based on the theoretical analysis of their temperature-dependent CD changes [60]. The results revealed a significant contribution of the charged pendant units to remarkable amplification of the helical chirality due to the reduction of the helical reversal population.

**Fig. 5:**
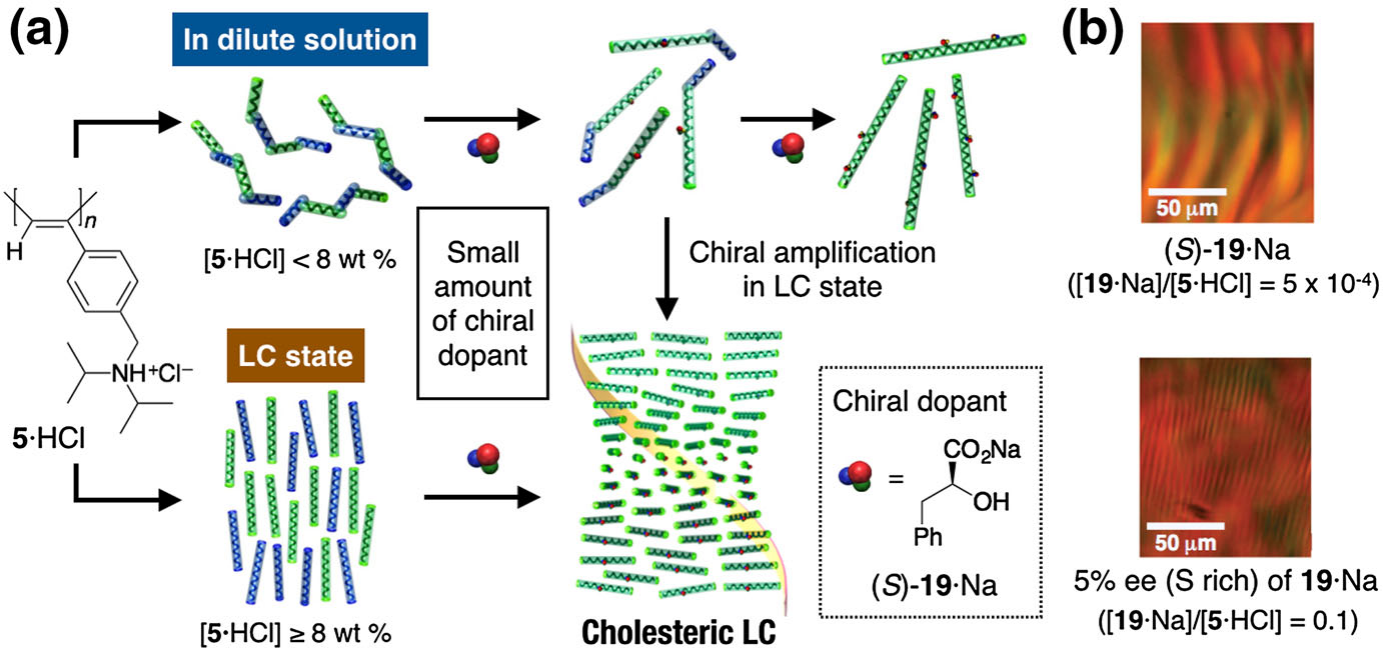
Schematic illustration of hierarchical amplification of macromolecular helicity in **5**·HCl by nonracemic dopants in dilute solution and LC state (**a**). Polarized optical micrographs of cholesteric LC phases of **5**·HCl (20 wt%) in the presence of 0.0005 equiv. of (*S*)-**19** (*top*) and 5% ee (*S* rich) of **19** (0.1 equiv.) in water (*bottom*) (**b**) (Reprinted with permission from [61]. Copyright 2006 American Chemical Society)

Interestingly, **5**·HCl formed a lyotropic, nematic LC in concentrated water (>8 wt%) (Fig. 5) [59, 61]. The addition of a tiny amount of chiral acids such as (*S*)-**19**·Na converted the nematic LC phase to the cholesteric counterpart to show a characteristic fingerprint texture due to the formation of the one-handed helical conformation in the polymer backbone of **5**·HCl. This liquid crystallinity of **5**·HCl originates from its main-chain stiffness in water as supported by its long persistence length (*q*) of 26.2 (nematic LC) and 28 nm (cholesteric LC) in the LC state [61]. The negatively charged polyelectrolyte **2b**·Na also formed a lyotropic LC phase in concentrated water (20–25 wt%) [58]. The polyelectrolyte characteristics of **2b**·Na and **5**·HCl seem to be essential for the appearance of the LC phase in water because the neutral **2** and **5** showed no LC phase in organic solvents. In the LC state, the helix-sense excess of **5**·HCl induced by chiral acids in dilute solution was further amplified through self-assembly into a cholesteric LC (Fig. 5a) [59, 61]. For example, the cholesteric helical pitch of **5**·HCl reached an almost constant value by the addition of 0.05 equiv. of (*S*)-**19**·Na, while in dilute solution, at least 0.3 equiv. of (*S*)-**19**·Na was necessary to induce the full CD. Interestingly, well-defined fingerprint cholesteric LC patterns were observed for the **5**·HCl in the LC state even in the presence of 5 × 10^−4^ equiv. of (*S*)-**19**·Na or 0.1 equiv. of nonracemic **19**·Na with 5% ee ((*S*) rich) (Fig. 5b). Direct comparison of the helical sense excess of the polymer chain in dilute solution with that in the cholesteric LC state clearly revealed that the macromolecular helicity induced in **5**·HCl by chiral dopants in dilute solution is further amplified in the LC state [61]. As observed in the LC polyisocyanates by Green et al. [62], the number of helix reversals existing between the right- and left-handed helical segments of the polymer may decrease in the LC state when compared with that in dilute solution because the kinked helix reversals are considered to hinder the close parallel packing of helical polymer chains in the LC state (Fig. 5a). The most plausible helical structure of **5**·HCl was determined to be a 23 U/10 turn (23/10) helix by the X-ray diffraction (XRD) analysis of the uniaxially oriented film of **5**·HCl prepared from its LC sample [61].

**Fig. 6:**
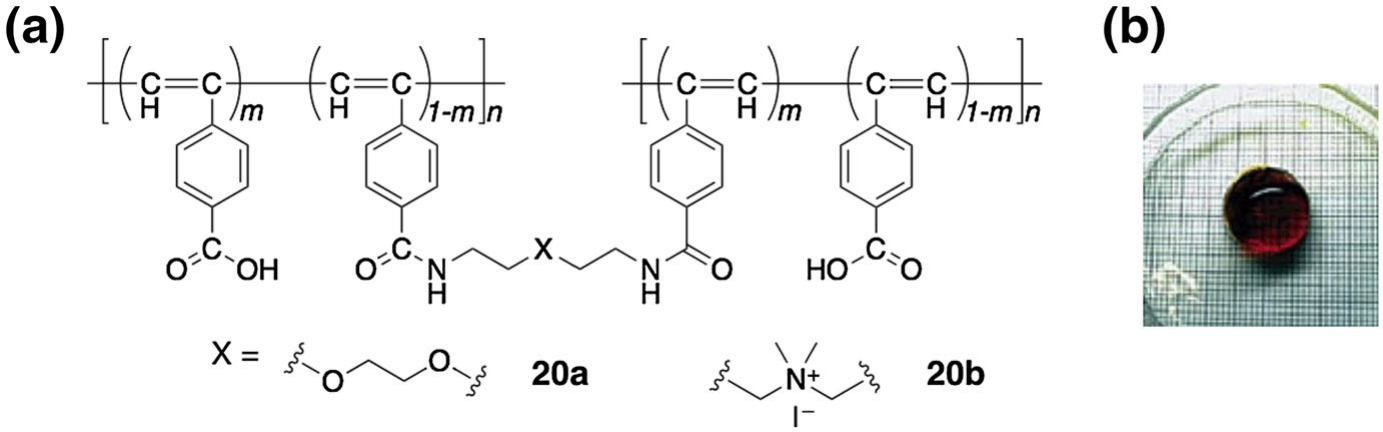
Chirality-responsive poly(phenylacetylene) gel (**20**) and a photograph of the gel

The macromolecular helicity induction in poly(phenylacetylene)s via noncovalent chiral interactions can be also attained in the gel state. Poly(phenylacetylene)-based gels (**20**) bearing carboxy pendants, which were prepared by the copolymerization of (4-carboxyphenyl)acetylene with a bis(phenylacetylene) used as the cross-linker or by the cross-linking reaction of the polymer **1** with a diamine, respectively, were the first chirality responsive gels (Fig. 6) [63]. These functional gels formed a predominantly one-handed helical conformation upon complexation with optically active amines in DMSO as well as in alkaline water accompanied by swelling, and the gels showed an ICD in the absorption region of the polymer backbone similar to that in the solution of **1**.

**Fig. 7:**
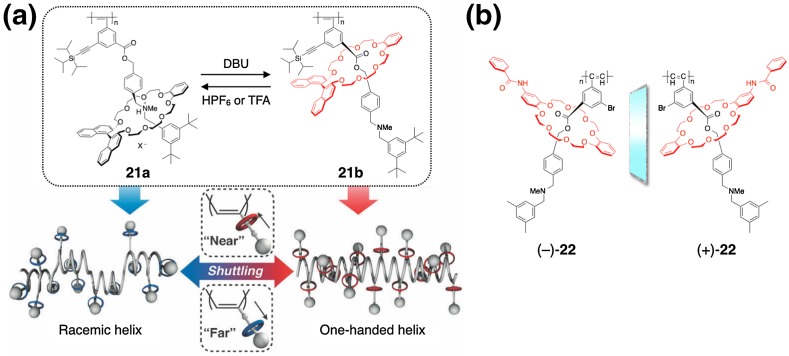
Schematic illustration of control of helical conformation of **21** by the positional switching of the optically active wheel component in the pendant rotaxane unit (**a**). Structures of poly(phenylacetylene)s bearing a planar chiral rotaxane pendant (**22**) (**b**) (Reprinted with permission from [65]. Copyright 2011 The Royal Society of Chemistry)

Takata et al. reported a unique methodology to control the helical conformation of a poly(phenylacetylene) derivative (**21**) possessing a rotaxane-based pendant, in which the positional switching of the chiral wheel component was demonstrated to work like a molecular machine (Fig. 7) [64, 65]. **21a** existed an almost racemic mixture of both helical conformations because the chiral wheel components were located around the *tert*-ammonium groups far from the polymer backbone and unable to induce a helix-sense bias (Fig. 7a). However, when the *tert*-ammonium groups were neutralized in situ by the addition of a base, such as 1,8-diazabicyclo[5.4.0]undec-7-ene (DBU), the pendant chiral wheels approached the ester groups close to the polyacetylene backbone (**21b**), thereby inducing a predominantly one-handed helical conformation as confirmed by the appearance of a clear ICD in the polymer backbone region. By the further addition of an acid, such as trifluoroacetic acid (TFA), **21b** returned to a racemic helical **21a**, and this conformational switching process can be repeated by sequential treatment with acid and base. A similar macromolecular helicity control has also been achieved by using analogous poly(phenylacetylene)s possessing an optically active rotaxane pendant with a planar chirality (**22**) (Fig. 7b) [66].

**Fig. 8:**
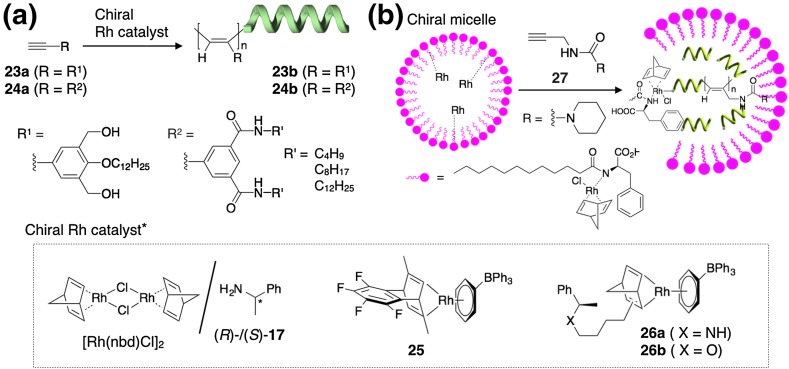
Schematic illustration of helix-sense-selective polymerization of achiral phenylacetylenes (**23**, **24**) with chiral Rh catalysts (**a**) and that of an achiral acetylene (**27**) in a chiral micelle (**b**) (Reprinted with permission from [72]. Copyright 2011 Willey-VCH)

Aoki et al. found that the polymerization of an achiral phenylacetylene (**23a**) possessing two hydroxy groups at the *meta* positions of the pendant phenyl ring with a rhodium catalyst such as [Rh(nbd)Cl]_2_ in the presence of (*S*)- or (*R*)-**17** produced an optically active poly(phenylacetylene) (**23b**) (Fig. 8a) [67, 68]. The obtained **23b** exhibited an intense ICD in the polymer backbone region in chloroform because of a predominantly one-handed helical conformation stabilized by intramolecular hydrogen bonds between the adjacent pendant hydroxy groups, demonstrating the first example of the helix-sense-selective polymerization of achiral phenylacetylene monomers. Therefore, the ICD intensity significantly decreased upon the addition of the increasing amount of DMSO and then disappeared. The polymerization of an analogous phenylacetylene (**24a**) also proceeded in a helix-sense-selective way to afford the corresponding optically active helical polymers (**24b)** with an excess handedness [69]. The polymerization of **23a** with optically active Rh complexes (**25**, **26**) also produced optically active helical polyacetylenes [70, 71] ]. Different from dynamic helical polyacetylenes (see Fig. 1 for example) whose preferred-handed helical structures are determined through noncovalent chiral interactions under thermodynamic control, the present optically active polyacetylenes with an excess one-handedness (**23b**, **24b**) were produced during the polymerization of monomers with chiral catalysts under kinetic control.

Deng et al. utilized chiral micelles consisting of d- or l-dodecylphenylalanine coordinated to [Rh(nbd)Cl]_2_ in which achiral *N*-propargylamides **27** was polymerized in a helix-sense-selective manner, thus producing optically active helical polyacetylenes, whose optical activity was maintained in chloroform after isolation from the micelles [72]. The intramolecular hydrogen bonds between the adjacent pendant urea groups as well as steric repulsions of the pendent groups may stabilize the helical conformation kinetically produced during the polymerization in the chiral micelles as evidenced by the disappearance of their optical activities in a highly polar solvent such as DMF (Fig. 8b).

Akagi et al. succeeded in synthesizing helically twisted polyacetylene fibrils with either a left- or right-handed screw direction by the polymerization of acetylene with Ziegler-Natta catalysts in chiral nematic LC phases [73].

## Memory of Macromolecular Helicity Induced in Polyacetylenes

**Fig. 9:**
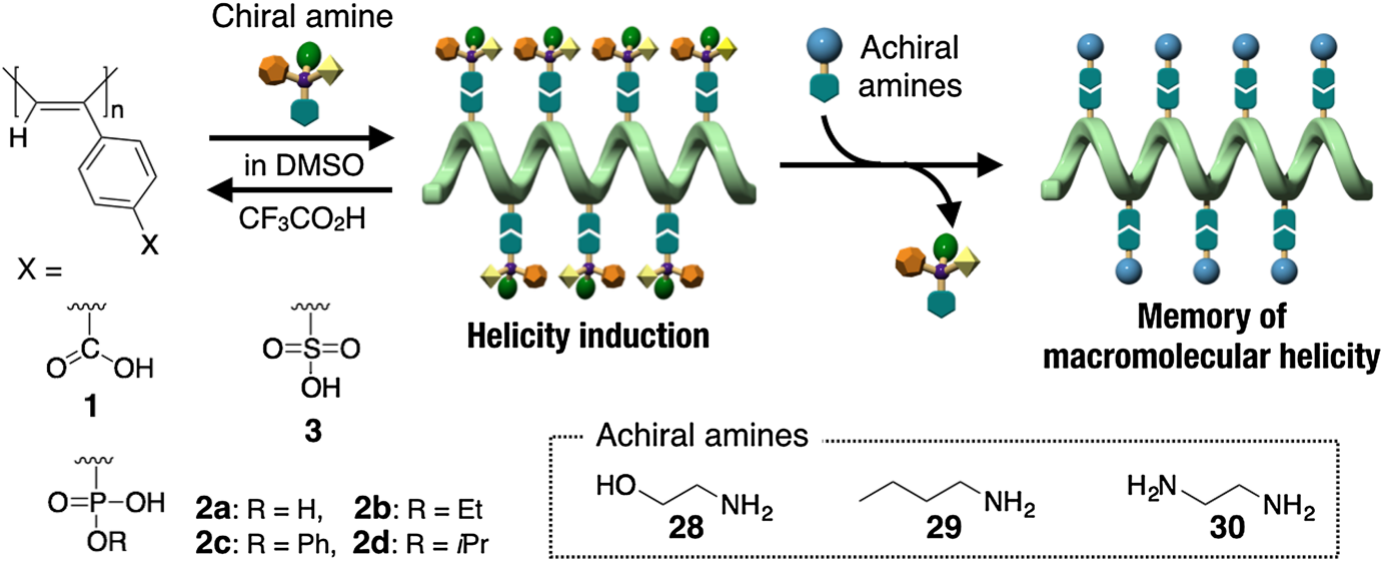
Schematic illustration of a helicity induction with an excess handedness in polyacetylenes (**1**–**3**) with chiral amines and memory of the macromolecular helicity through replacement with achiral amines

The predominantly one-handed helical conformations induced in poly(phenylacetylene)s (**1**–**3**) through noncovalent bonding interaction with optically active amines are immediately changed to racemic ones when the optically active amines are removed from the polymers using a stronger acid such as TFA because the original helical conformations of these polymers are dynamic in nature. However, the induced macromolecular helicity in **1**–**3** can be retained, namely “memorized” in solution when the optically active amines are replaced by achiral amines or diamines, such as **28** and **29** for **1** and **30** for **2** and **3** (Fig. 9) [43, 49, 74, 75]. For example, polymer **1** formed a preferred-handed helical conformation upon complexation with (*R*)-**18** in DMSO to show an intense ICD in the polymer backbone region. The ICD of **1** induced by (*R*)-**18** was almost retained after the polymer had been isolated as the complex with an achiral amino alcohol **28** by size exclusion chromatography (SEC) fractionation of the **1**–(*R*)-**18** complex using DMSO containing **28** (0.8 M) as the mobile phase [74]. The memory efficiency assisted by **28** was estimated to be 87% by comparison of the ICD intensity of the isolated **1**–**28** complex just after the SEC fractionation with that of the **1**–(*R*)-**18** complex. The memory efficiency was significantly influenced by the achiral amine structures used for the helicity memory. The macromolecular helicity memory persisted for a long time, over 2 years for the **1**–**28** complex. The mechanism of the preferred-handed helicity induction and its memory effect in **1** has been thoroughly investigated using various spectroscopic methods including absorption, CD, and IR spectroscopies. It was revealed that a preferred-handed helicity is cooperatively induced in **1** via the ion pair formation of the carboxy pendants with optically active amines, and the free ion formation plays a key role in the maintenance of the helicity memory after the chiral amine has been replaced with achiral amines. Through the free ion formation of the complex, the helix inversion of the polymer backbone can be efficiently hampered by the intramolecular electrostatic repulsion between the adjacent pendant carboxylate ions with negative charges [75]. As a result, the original dynamic helical conformations of **1**–**3** are transformed into metastable static ones assisted by the achiral amines or diamines.

**Fig. 10:**
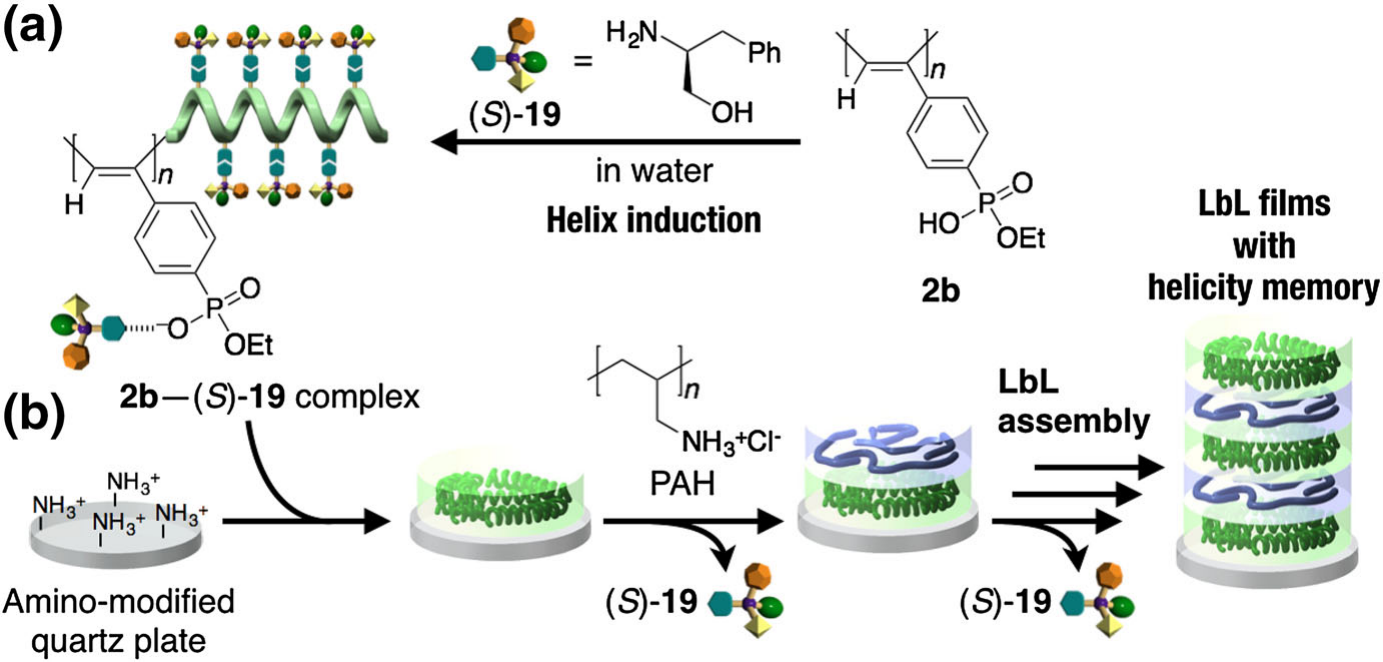
Schematic illustration of a macromolecular helicity induction through the LbL self-assembly of a charged polyacetylene. A preferred-handed macromolecular helicity is induced in **2b** upon complexation with (*S*)-**19** in water (**a**). A negatively charged **2b** with induced macromolecular helicity can be LbL assembled with a positively charged PAH to give multilayer thin films with helicity memory on a quartz plate (**b**) (Reprinted with permission from [76]. Copyright 2005 The Royal Society of Chemistry)

Although macromolecular helicity induction in **1**–**3** with chiral guests is also possible in water as described in the previous section (see Sect. 2), the induced helicity was instantly lost in water when the optically active amines were removed and replaced with achiral ones. However, the macromolecular helicity memory in water was achieved by using a layer-by-layer (LbL) assembly technique. For example, alternate adsorption of a negatively charged helical poly(phenylacetylene) bearing phosphonate pendants **2b** with an excess handedness induced by a chiral amine (*S*)-**19** in water and a positively charged achiral polymer such as the hydrochloride of poly(allylamine) (PAH) on a positively charged quartz substrate gave an optically active multilayer thin film. The film showed an apparent ICD in the polymer backbone region. The ICD intensity of the thin film increased with the increasing number of the alternate adsorption cycle. Interestingly, the multilayer thin films contained no (*S*)-**19** used for the helicity induction in **2b** because of automatic removal of the chiral amine during the LbL assembly process (Fig. 10) [76], indicating the **2b** possesses the macromolecular helicity memory in the films. The resulting LbL assembled multilayer thin films will be applicable to the development of novel chiral materials for separation of enantiomers and asymmetric catalysis by further modification of the polymers with specific metals.

**Fig. 11:**
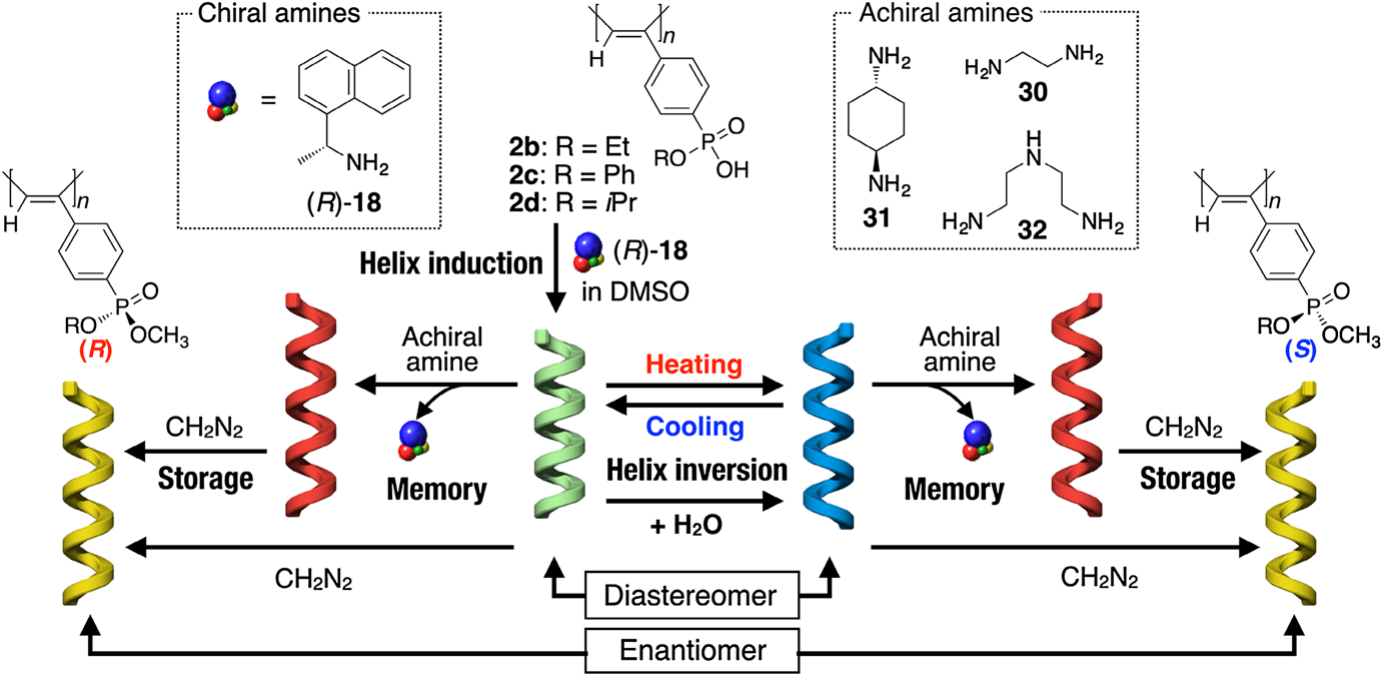
Schematic illustration of a helicity induction with an excess handedness in polyacetylenes (**2b**–**d**) with (*R*)-**18**, helix inversion by solvent and/or temperature, memory of the diastereomeric macromolecular helices assisted by achiral amines (**30**–**32**), and storage of the induced helicity and the macromolecular helicity memory through enantioselective esterification with diazomethane (Reprinted with permission from [52]. Copyright 2015 American Chemical Society)

The preferred-handed helicity induction in dynamically racemic helical polymers via noncovalent chiral interactions followed by the helicity memory provides one of the useful methods for producing optically active helical polymers. Since the chirality of the enantiomeric guests used determines the helical sense of the polymers, the opposite enantiomeric guest is requisite for producing the opposite-handed helical polymers with helicity memory. The achiral solvent-induced or temperature-dependent inversion of the macromolecular helicity is one of the most characteristic features of dynamic helical polymers. Taking advantage of this feature, it has been revealed that both enantiomeric right- and left-handed helices with helicity memory that are mirror images of each other can be produced from dynamic helical poly(phenylacetylene)s using a single enantiomer as a helicity inducing agent. This phenomenon can be regarded as “dual memory” (Fig. 11) [46, 52]. For example, the predominant helical senses of poly(phenylacetylene)s possess prochiral phosphonic acid monoester pendants (**2b**–**d**) induced by the chiral amine (*R*)-**18** in DMSO inverted by the addition of water (5–20 vol%), as supported by the inversion of the Cotton effect signs. The resulting right- and left-handed helices are diastereomers to each other because they complex with the same enantiomeric amine as the helicity inducer. However, the subsequent macromolecular helicity memory assisted by achiral amines such as **30**–**32**, which replace the (*R*)-**18**, successfully produced the corresponding enantiomeric helices, exhibiting the perfect mirror image CD spectral patterns together with identical absorption spectra.

The helicity memory, however, gradually disappears because of its inherently dynamic characteristics. Interestingly, such a dynamic helicity memory could be stored by generating a phosphorus stereogenic center with an optical activity in the pendants through asymmetric methyl esterification of the prochiral phosphonic acid monoester pendants using diazomethane [52, 77]. The esterification reaction proceeded in an enantioselective way because of the chirality transfer from the preferred-handed macromolecular helicity induced and/or memorized in the polymer backbone of **2**, and the resulting optically active phosphorus pendant groups enable the polymer backbone to take a preferred-handed helical conformation (Fig. 11). The structures of the phosphonic acid monoester pendants in **2** largely affected the enantioselectivity during the methyl esterification, among which **2d** afforded the highest enantioselectivity of 33% ee. The enantioselectivity is rather low, but the helix-sense excess in the polymer backbone can be further effectively biased because of the characteristic chiral amplification in dynamic helical polymers to reach 84% ee at −50 °C [52].

**Fig. 12:**
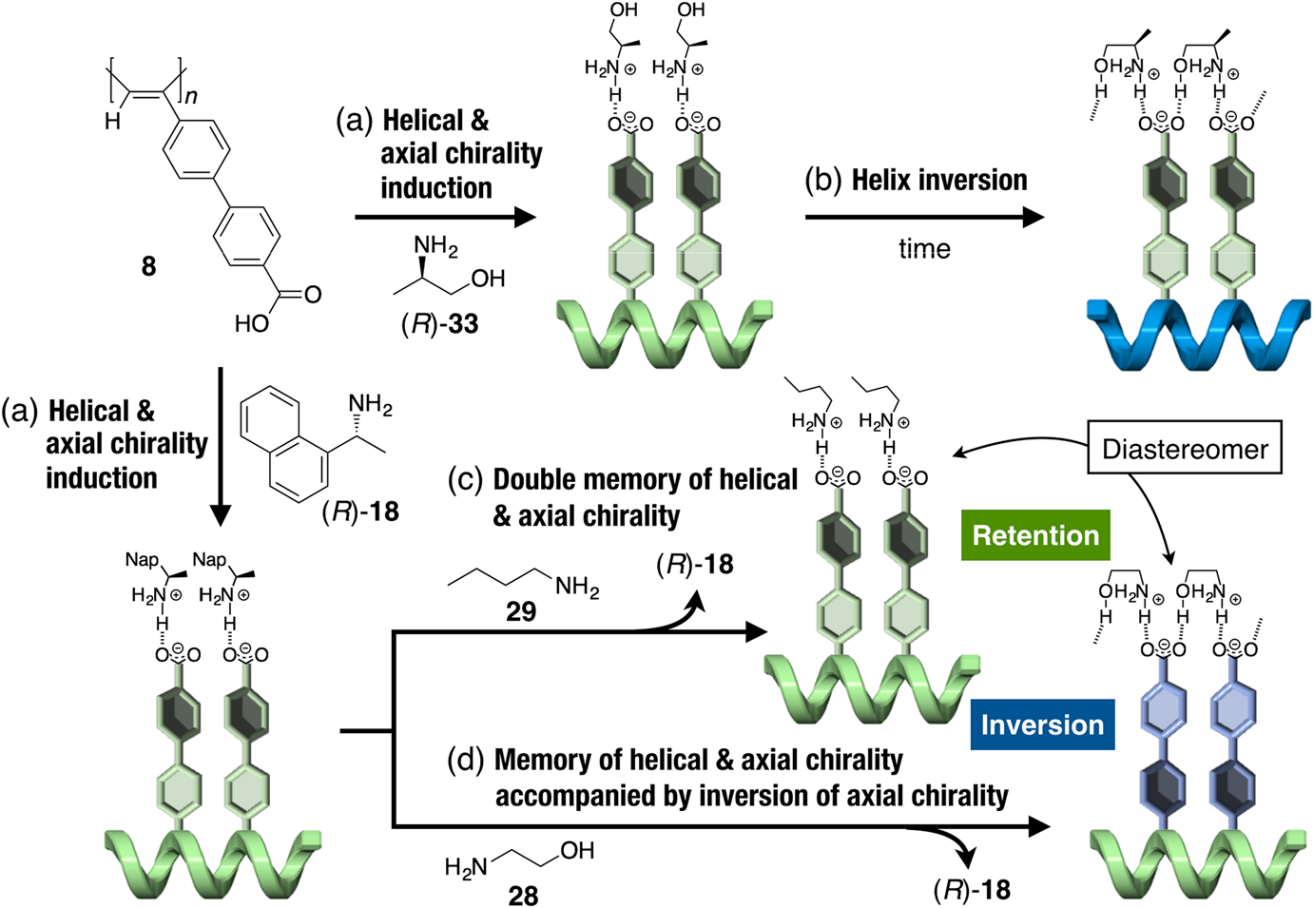
Schematic illustration of induction of a preferred-handed macromolecular helicity and axial chirality of the biphenyl pendants in **8** upon complexation with chiral amines (*R*)-**18** and (*R*)-**33** (*a*), macromolecular helicity inversion of **8** with time after complexation with (*R*)-**18** (*b*), memory of the macromolecular helicity and axial chirality of the biphenyl pendants assisted by interaction with achiral **29** (*c*) and **28** (*d*). The helicity memory of **8** with **28** is accompanied by inversion of the axial chirality of the biphenyl pendants followed by memory of the inverted biphenyl chirality (*d*) (Reprinted with permission from [51]. Copyright 2008 Wiley-VCH)

Introduction of a biphenyl substituent with dynamic axial chirality instead of a phenyl group to a polyacetylene backbone as the pendant endowed the helical polyacetylenes with unique features in noncovalent helicity induction and/or the subsequent memory effect [51, 78]. A *cis*-*transoidal* polyacetylene (**8**) that carries a carboxybiphenyl group as the pendant folded into a predominantly one-handed helical conformation upon complexation with chiral amines, such as (*R*)-**18** and (*R*)-**33**, in DMSO, which was accompanied by the formation of an excess single-handed, axially twisted conformation in the biphenyl pendants (Fig. 12a) [51]. When **8** was complexed with (*R*)-**33**, the complex showed a unique time-dependent inversion of the macromolecular helicity in the polymer backbone (Fig. 12b). The complexation of **8** with (*R*)-**33** through acid-base interactions seems to bias the twist sense of the axially twisted conformation in the biphenyl units to determine the initial helix sense of the polymer backbone in **8** (Fig. 12a). Then, the following hydrogen bond formation between the hydroxy group of (*R*)-**33** and an adjacent carboxy pendant of **8**, which favors the opposite helix sense of the polymer backbone, induces the inversion of the macromolecular helicity (Fig. 12b). Both the induced macromolecular helicity in the polymer backbone and the twisted biphenyl chirality in the pendants were memorized after the chiral amine (*R*)-**18** had been removed and replaced with an achiral amine **29** (Fig. 12c). Interestingly, the macromolecular helicity memory of **8** using achiral amino alcohol **28** as a chaperoning compound was accompanied by the inversion of the axial chirality of the biphenyl groups, thus showing a significant change in the CD spectral pattern (Fig. 12d).

**Fig. 13:**
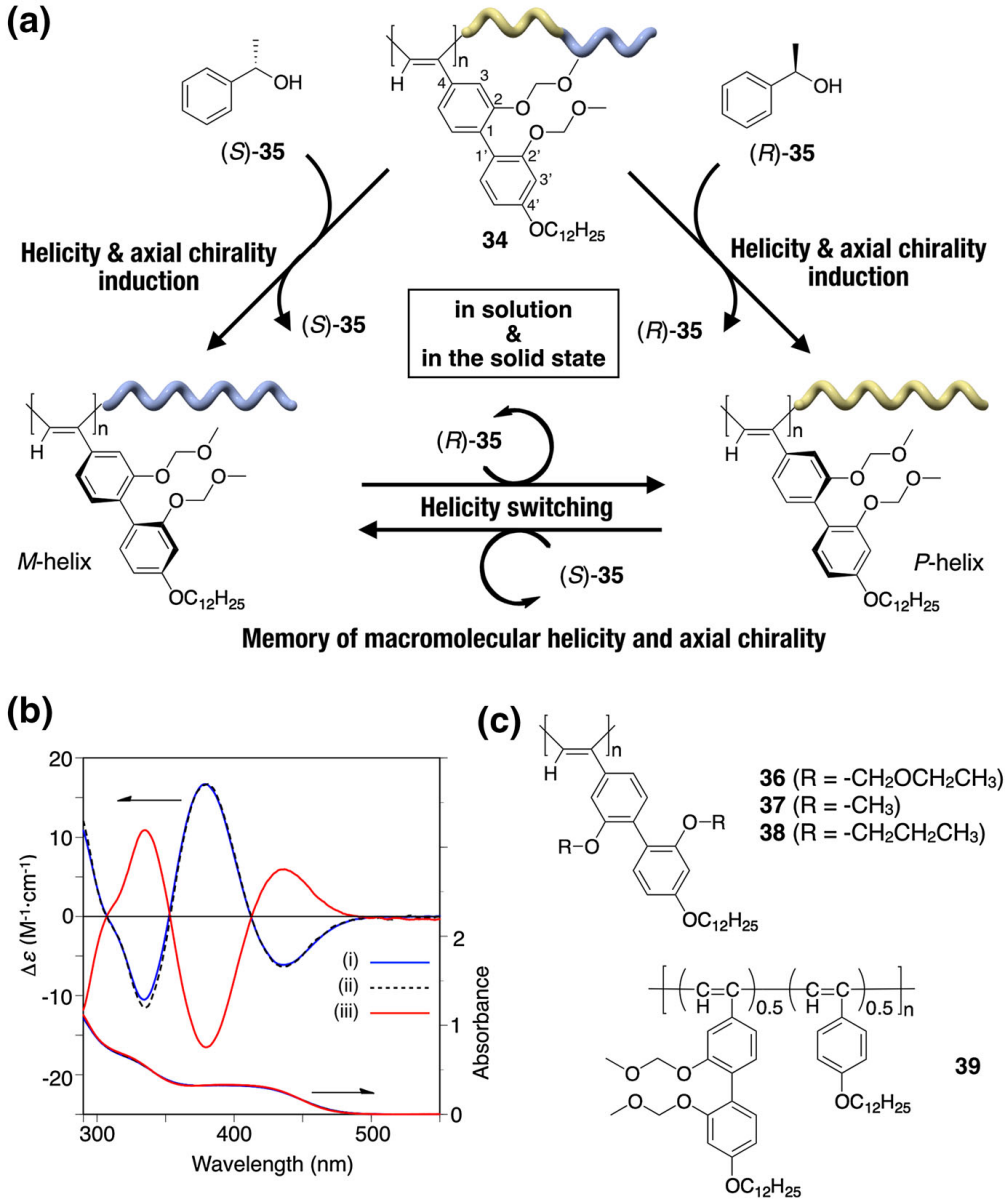
Schematic illustration of a switchable induction and memory of macromolecular helicity along with that of the pendant axial chirality in **34** in the solid state as well as in solution. Predominantly one-handed helical conformation and axial chirality in the biphenyl pendants are induced in **34** through noncovalent interactions with a nonracemic **35**, and both of them are automatically memorized after complete removal of **35**. The helical handedness and axial twist sense of **34** are switched reversibly upon interaction with the opposite enantiomeric alcohol in the solid state (**a**). CD and absorption spectra of **34** in the presence of (*S*)-**35** in *n*-hexane at 25 °C (*i*) and those of the isolated **34** dissolved in *n*-hexane at −10 °C after soaking in (*S*)-**35** at 25 °C for 6 h in the solid state followed by isolation (*ii*) and subsequent soaking in (*R*)-**35** at 25 °C for 6 h (*iii*) (**b**). Structures of the copolymer (**39**) and analogous polyacetylenes bearing 2,2′-bisphenol-derived pendants (**36**–**38**) (**c**) (Reprinted with permission from [78]. Copyright 2014 Nature Publishing Group)

A very unique switchable helicity induction and subsequent memory of the induced macromolecular helicity, which is attainable both in solution and in the solid state, has been reported on a *cis*-*transoidal* polyacetylene bearing 2,2′-biphenol-derived pendants, when the phenolic hydroxy groups are modified with methoxymethoxy (OMOM) groups (**34**) (Fig. 13) [78]. Most previously reported examples of noncovalent helicity induction in optically inactive polymers were performed in solution, and those in the solid state are quite rare [79–81]. Moreover, in this system, the replacement of the chiral inducers by achiral chaperoning compounds is totally unnecessary for the helicity memory. For example, after soaking in liquid (*S*)-**35** at 25 °C, the isolated **34** dissolved in *n*-hexane at −10 °C showed a clear ICD [Fig. 13b(ii)], whose pattern and intensity were almost the same as those observed in *n*-hexane in the presence of (*S*)-**35** (Fig. 13b(i)). Subsequent soaking in (*R*)-**35** resulted in perfect mirror image ICDs [Fig. 13b(iii)]. Since **34** is completely insoluble in **35**, these results clearly demonstrate that a predominantly one-handed helicity can be induced in the polymer backbone of **34** even in the solid state just by allowing **34** to interact with optically active liquid **35**, and the induced helicity is automatically memorized without replacement by achiral compounds. Furthermore, an excess single-handed, axially twisted conformation was simultaneously induced and automatically memorized in the biphenyl pendants during the macromolecular helicity induction in **34** through noncovalent interaction with the optically active **35**. This speculation was supported by the appearance of an intense vibrational circular dichroism (VCD) signal in the absorption region due to the pendant OMOM groups. A similar helicity induction and memory effect were observed for **36**-bearing ethoxymethoxy groups instead of OMOM groups, while the analogous **37**-bearing methoxy groups and **38**-carrying propoxy groups instead of OMOM groups exhibited no memory effect and no helicity induction, respectively, under similar conditions (Fig. 13c). Moreover, the copolymer **39** prepared by the copolymerization of **34** with a phenylacetylene having the identical alkoxy pendant almost lost the memory effect (Fig. 13c). Based on these results, both the biphenyl pendants and the alkoxymethoxy substituents at its 2- and 2′-positions seem to play an important role in achieving this unique helicity induction and subsequent memory effect, where the biphenyl pendants possessing alkoxymethoxy groups probably act as a geared molecular brake that can prevent racemization of the helical main chain. These unusual features of switchable helicity induction and subsequent memory effect in the solid state observed for **34** were utilized for developing an unprecedented switchable chiral stationary phase (CSP) for high-performance liquid chromatography (HPLC) in which the elution orders of the enantiomers can be reversibly inverted at will (see Sect. 4) [78].

**Fig. 14:**
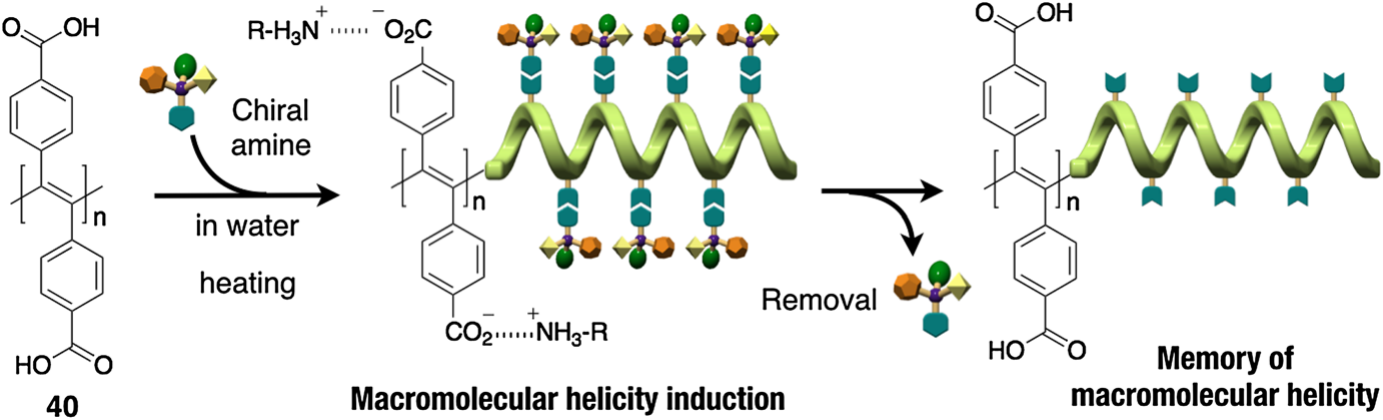
Schematic illustration of a helicity induction with an excess handedness in **40** in the presence of chiral amines upon heating and subsequent memory of the macromolecular helicity after complete removal of the chiral amines

A similar helicity memory free of assistance by achiral chaperoning compounds has been observed in an optically inactive poly(diphenylacetylene) (PDPA) derivative bearing carboxy pendants (**40**), although thermal annealing is necessary for the helicity induction process [82]. When **40** was complexed with optically active amines in water and then annealed at 90 °C, a preferred-handed helical conformation was induced in the polymer backbone, which was then automatically memorized after complete removal of the optically active amines at ambient temperature (Fig. 14). The macromolecular helicity induced in **40** was maintained even after conversion of the pendant carboxy groups into methyl esters by the reaction with trimethylsilyldiazomethane. Each pendant phenyl ring of the **40** is highly restricted from free rotation as confirmed by its temperature-dependent ^1^H NMR spectral changes in a DMSO-*d*_6_-D_2_O mixture (20/1, v/v), where the signals attributed to the protons at the *ortho*- and *meta*-positions on each phenyl ring were observed as two largely separated sets of peaks, respectively, and these peaks coalesced into single ones at 130 (*ortho*) and 110 (*meta*) °C. The free energy of activation (∆*G*) for the rotation barrier of the pendant phenyl rings was calculated to be 78.5 and 78.3 kJ/mol, respectively, on the basis of the coalescence temperatures. This restricted rotation of the pendant phenyl rings with a considerably high energy seems to play an important role for achieving the helicity memory in **40** without the assistance of the achiral compounds.

Lee et al. reported that an optically inactive PDPA without functional groups in the pendants (**41**) can be transformed into an optically active one by dissolving in optically active limonene followed by thermal annealing. The observed optical activity has been considered because of a formation of a helical arrangement of the pendant phenyl rings with a preferred-handed screw sense along the polymer backbone through noncovalent interactions with the chiral limonene [83]. After isolation of the polymer, the recovered polymer dissolved in achiral solvents maintained its optical activity, which means the memory of the helical chirality induced in **41** by the chiral solvent [84]. The same group also reported that an optically active helical **41** can be obtained by the helix-sense-selective polymerization of the corresponding monomer in optically active *α*-pinene by using TaCl_5_–*n*Bu_4_Sn as a catalyst [85]. These results indicate that **41** can act as both static and dynamic helical polymers because its helix inversion barrier may be between those of the two types of helical polymers, dynamic and static ones.


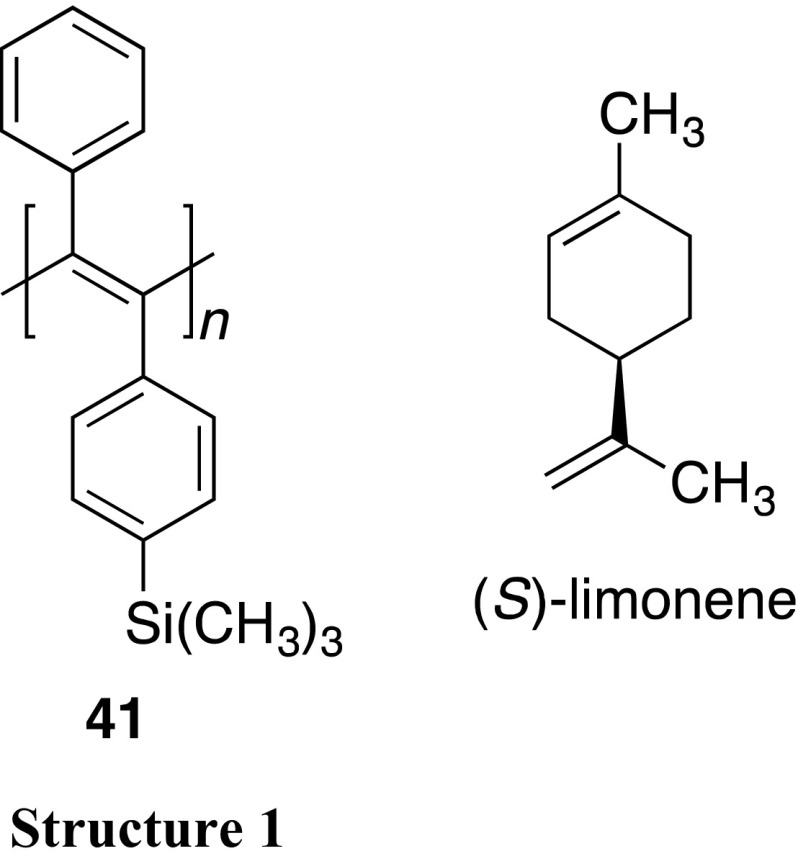


## Application of Helical Polyacetylenes as Chiral Materials

Inspired by the sophisticated functions of biological helices, development of helical polymer-based advanced materials has gathered much attention. Until now, several optically active helical polymers with a controlled helix sense have been applied as chiral materials, such as enantioselective adsorbents and catalysis. In this section, we describe some successful examples of the application of one-handed helical polyacetylenes constructed by macromolecular helicity induction and/or the memory effect.

Separation of enantiomers by HPLC using a CSP is one of the most popular and effective methods not only for analyzing the enantiomeric composition of chiral compounds but also for obtaining pure enantiomers [86, 87]. The one-handed helical PTrMA synthesized by the helix-sense-selective polymerization of the corresponding achiral monomer can resolve various racemic compounds including chiral drugs when used as a CSP for HPLC and has been commercialized [88–90]. This is the first example of the practical application of synthetic helical polymers as chiral materials. Since this discovery, a number of helical polymer-based CSPs have been developed because the one-handed helical structure appears to play an essential role in achieving the high-resolution abilities as CSPs for HPLC [1, 4, 87, 91, 92]. Helical polyacetylenes possessing optically active pendants (**42**–**44**) also have been prepared, and some of them exhibit good chiral recognition abilities as CSPs for HPLC because of their preferred-handed helical conformation [26, 93–101].


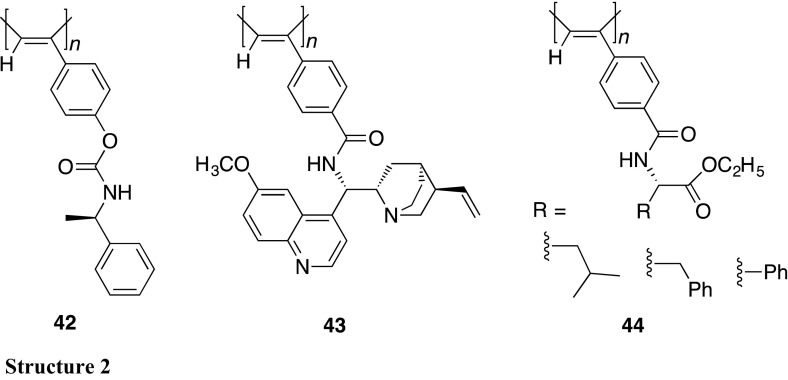


In enantiomer separation by HPLC using CSPs, the elution order of the enantiomers often becomes an important issue in both the analytical and preparative modes. In the analytical mode, the elution of the minor enantiomer should precede that of the major one because this elution order can lead to enhancement of the detection limit and the accuracy of the quantification. In the preparative mode, the desired enantiomer can be obtained with a higher optical purity if the target enantiomer is eluted first because there is a large possibility that a part of the first-eluting enantiomer overlaps with the second one. Therefore, from the practical point of view, control of the elution order is highly demanded, particularly when the difference in the elution times of a pair of enantiomers is small. We usually use the CSPs composed of enantiomeric chiral materials to invert the elution order. The development of CSPs capable of inverting the elution order under identical chromatographic conditions remains very challenging.

**Fig. 15:**
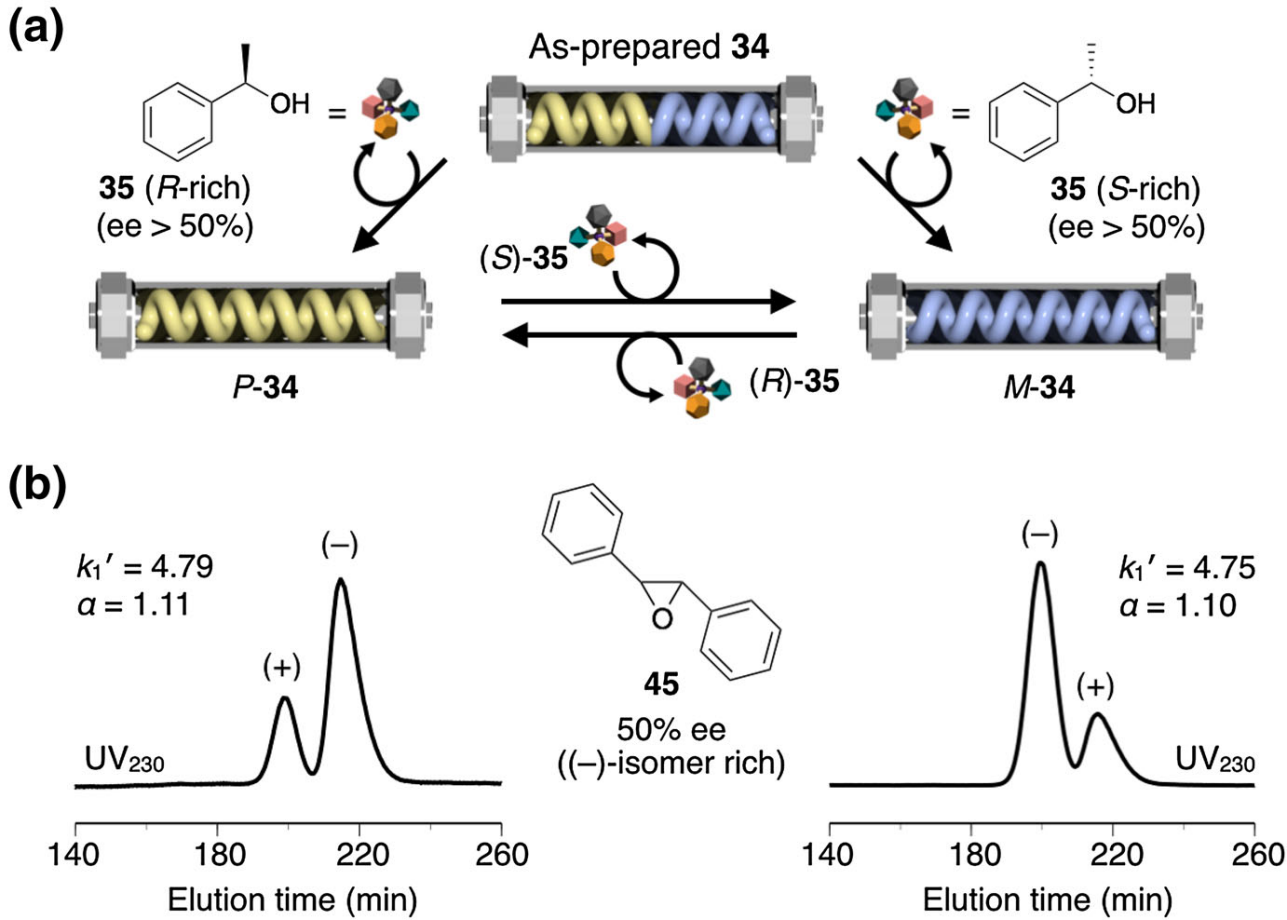
Schematic illustration of a CSP for HPLC capable of switching the elution order of enantiomers based on reversible switching and subsequent memory of the macromolecular helicity in **34** by alternative treatment with (*R*)- or (*S*)-**35** (ee > 50%) (**a**). Separation of 50% ee of **45** ((−)-isomer rich) on *P*-**34** (*left*) and *M*-**34** (*right*) at ca. 0 °C (eluent, methanol/H_2_O (75/25, v/v); flow rate, 0.025 ml/min) (**b**) (Reprinted with permission from [78]. Copyright 2014 Nature Publishing Group)

As described in the previous section (see Sect. 2), the *cis*-*transoidal* polyacetylene bearing 2,2′-biphenol-derived pendants **34** shows the remarkable features of switchable macromolecular helicity induction and subsequent memory in the solid state as well as in solution. These intriguing properties enabled us to develop an unprecedented switchable CSP for HPLC, whose helical chirality can be directly switched in the column by alternative passage of an eluent containing either an nonracemic (*R*)- or (*S*)-alcohol, leading to the reversible switching of the elution order of the enantiomers (Fig. 15) [78]. The as-prepared optically inactive **34** coated on porous silica gel was packed into a column, which was then treated with an acetone solution of (*R*)-**35** (50 vol%) to induce a preferred-handed helix in **34** followed by treatment with methanol to remove (*R*)-**35** completely. This procedure afforded the CSP composed of **34** with a right-handed helicity memory (*P*-**34**), and this *P*-**34**-based column almost completely resolved the 50% ee ((−)-isomer rich) of **45** with a separation factor (*α*) of 1.11, in which the minor (+)-enantiomer eluted first followed by the major (−)-one (Fig. 15b). The helicity of **34** was then inverted from the right- to left-handed helix by treating the *P*-**1**-based CSP with an acetone solution of the antipodal (*S*)-**35** (50 vol%) in the column. The following treatment with methanol produced the *M*-**34**-based CSP with an opposite helicity memory, which resolved the enantiomers of **45** with the opposite elution order, but virtually the same retention factor (*k*_1_) and *α* values as those observed for the *P*-**34**-based CSP (Fig. 15b). Thus, the switchable separation of enantiomers under the identical chromatographic condition was, for the first time, achieved based on noncovalent helicity induction and its memory effect in the optically inactive polyacetylene in the solid state [78].

However, the chiral recognition ability of **34** with helicity memory as a CSP for HPLC was not high because of the absence of effective interaction sites with the enantiomers. Introduction of a functional group at the 4′-position of the biphenyl pendant was found to contribute not only to improvement of the chiral recognition ability but also to the stabilization of the helicity memory. For example, an analogous polyacetylene **46** bearing an ester group at the 4′-position of the biphenyl pendant also showed a similar helicity induction and memory effect, and the macromolecular helicity memory of **46** was much more stably maintained than that of **34** [102]. In toluene at 25 °C, the half-life period (*t*_1/2_) of the macromolecular helicity memory in **46** (ca. 4 h) was more than 20-fold longer than that in **34** (<0.2 h). The intramolecular dipole–dipole interaction between the neighboring ester functional groups seems to contribute to the observed stabilization of the helicity memory. The **46**-based CSP with a helicity memory showed a better chiral recognition ability than that of **34** and efficiently resolved chiral binaphthyl compounds (**48**–**50**) and metal tris(acetylacetonato)s (**51**–**53**) probably because of an effective interaction between the ester groups of **46** and the enantiomers [102]. On the other hand, introduction of two ester (acetyloxy) groups at the 2- and 2′-positions of the biphenyl pendant instead of two OMOM groups resulted in destabilization of the helicity memory. Although a similar helicity induction and memory effect were also observed for **47**, the helicity memory of **47b** disappeared more quickly than that of **34** in methylcyclohexane [103]. However, in the solid state, the helicity memory of **47b** was maintained for an extremely long time, at least for 1 month at 25 °C as confirmed by no detectable decrease in the ICD intensity, which enabled us to utilize **47** as a CSP for HPLC. Although the **47a**-based CSP could not separate racemates **49**–**53** including the metal tris(acetylacetonato)s (**51**–**53**), which were separated on **46**, benzoin (**54**) and its analogs (**55**–**57**) as well as **48** and **45** were well resolved on **47a**, but not resolved on **46**. The difference in the chiral recognition abilities between **46**- and **47a**-based CSPs may be ascribed to the difference in the induced helical structures between **46** and **47a** as evidenced by the different ICD patterns in the polymer backbone regions from each other, which results in the helical arrangement of the pendants in a different helical array along the polymer backbone.


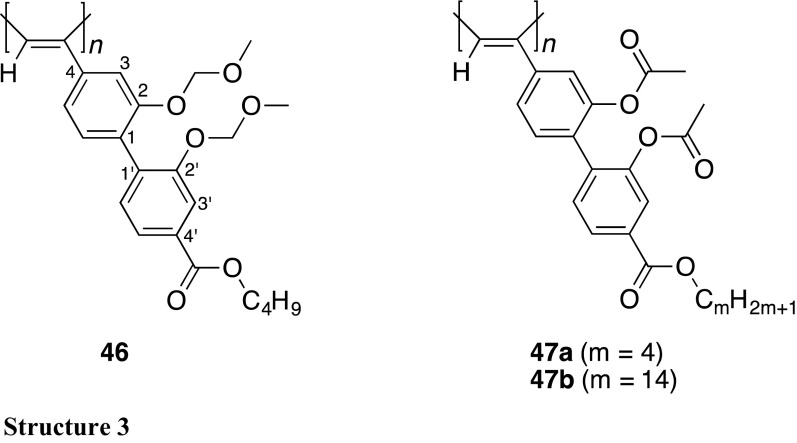
 
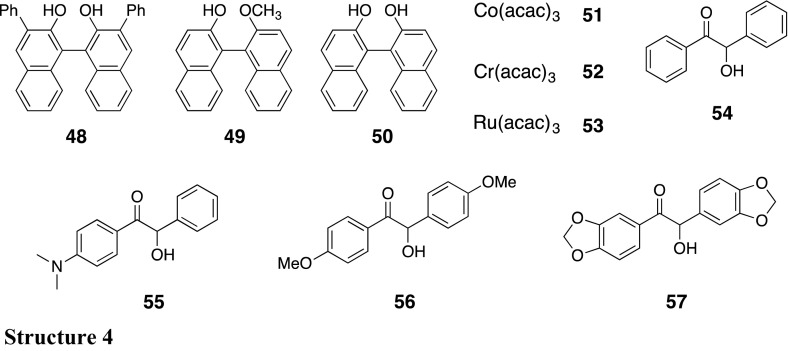


**Fig. 16:**
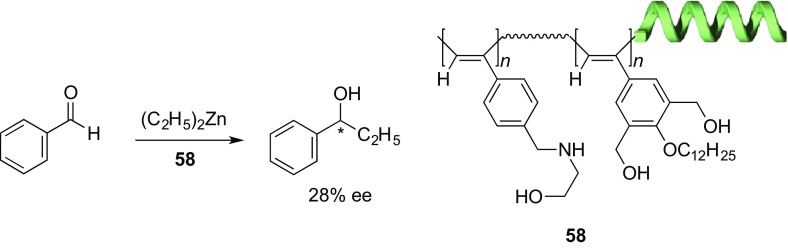
Asymmetric reaction using a one-handed helical polyacetylene (**58**) prepared by helix-sense-selective polymerization

Application of optically active helical polymers to asymmetric catalysts has been attracting much attention because of the intriguing synergistic effect of the helical chirality on the enantioselectivity, thereby leading to a more efficient asymmetric catalytic activity than that expected from the chiral pendant ligand itself attached to the polymer backbone [1, 4]. A number of helical polyacetylenes possessing optically active pendants have been used as the polymeric catalysts for various types of asymmetric reactions [104–113]. Among them, **43** bearing the chiral quinine residues through an amide linkage is one of the successful examples of helical polyacetylene-based asymmetric catalysts [111]. **43** showed a high level of enantioselectivity toward the asymmetric Henry reaction of 4-nitrobenzaldehyde with nitromethane, forming a product up to 94% ee, which is much higher than that catalyzed by the corresponding monomeric compound (28% ee) and the non-helical counterpart (18% ee), suggesting the importance of the helical chirality of **43** in the excellent enantioselectivity. One-handed helical poly(phenylacetylene)s possessing achiral amino alcohol moieties (**58**) synthesized by the helix-sense-selective copolymerization of the corresponding achiral acetylene monomers with a rhodium catalyst in the presence of optically active amines catalyzed the enantioselective diethylzinc addition to benzaldehyde, giving a moderate enantioselectivity (ca. 20–30% ee) (Fig. 16) [114]. This result clearly demonstrates that preferred-handed helical polyacetylenes composed of achiral monomer units can also function as a promising helical polymer-based asymmetric catalyst.

**Fig. 17:**
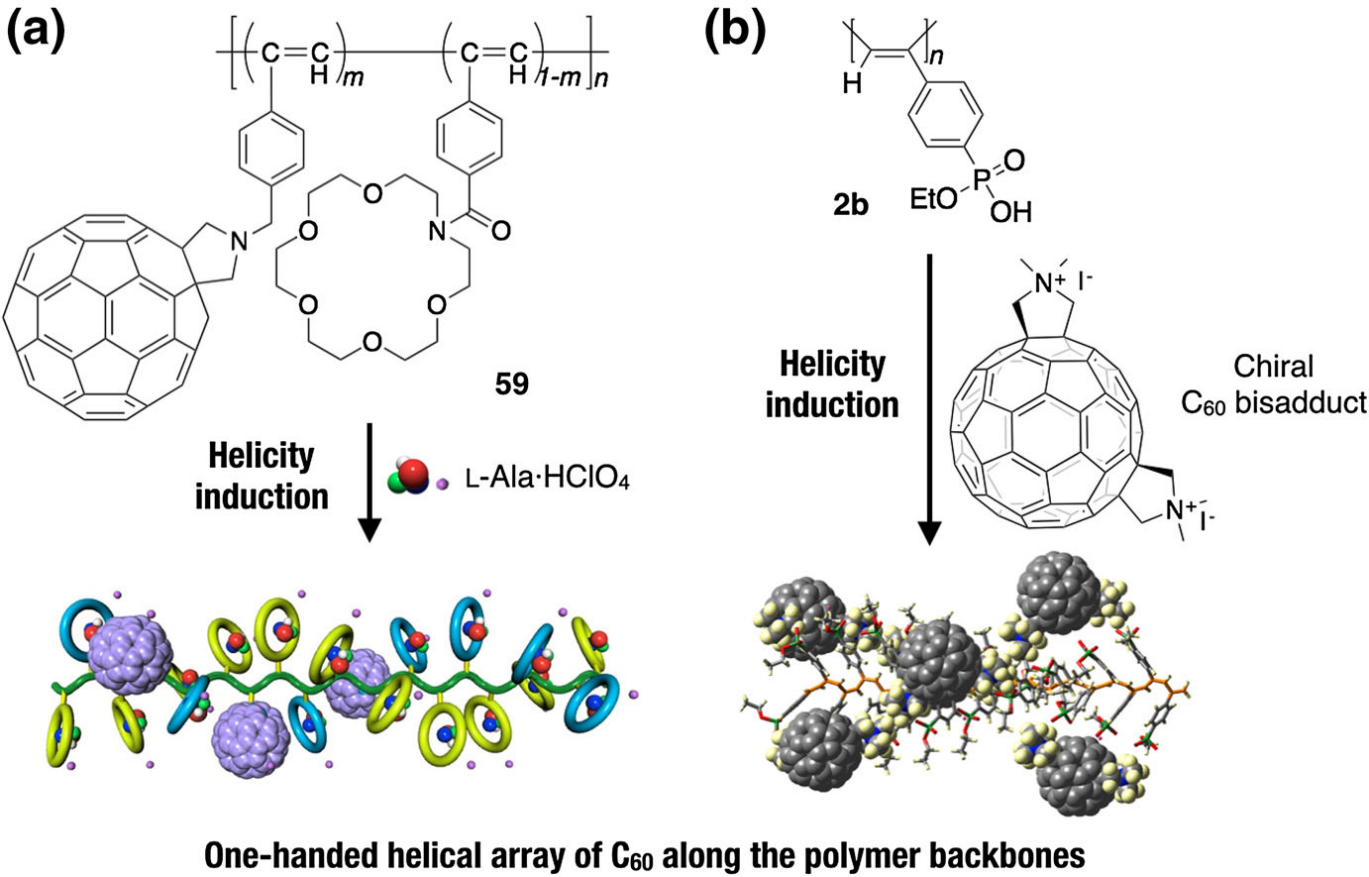
Schematic representation of predominantly one-handed helical arrangements of fullerenes along the polyacetylene backbones assisted by macromolecular helicity induction in polyacetylenes (**59** and **2b**) via noncovalent interaction with chiral compounds (Reprinted with permission from [115] and [116]. Copyright 2004 The Royal Society of Chemistry and Copyright 2004 American Chemical Society)

**Fig. 18:**
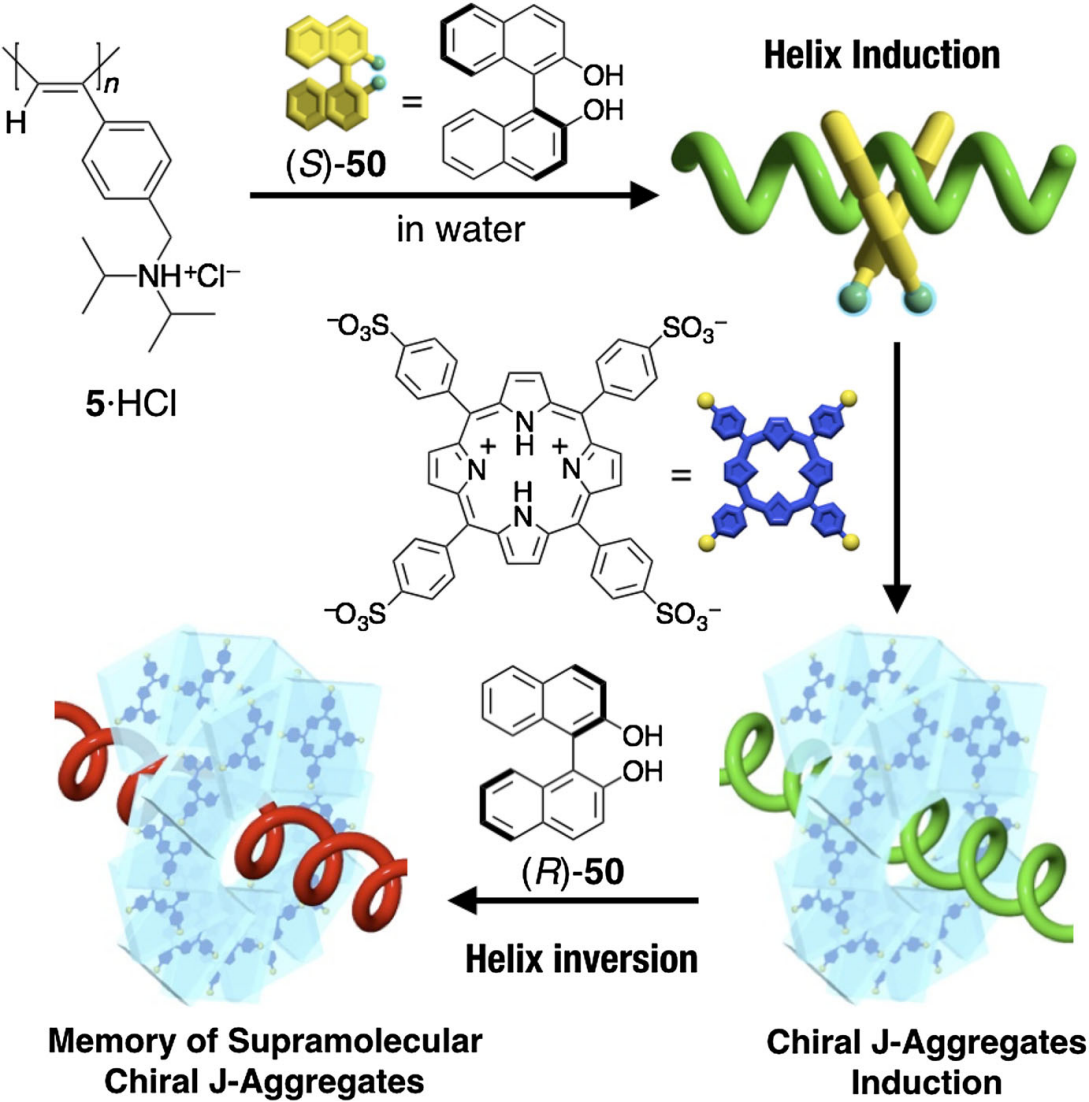
Schematic representation of a helicity induction with an excess handedness in water-soluble **5**·HCl with (*S*)-**50**, subsequent formation of supramolecular helical J-aggregates of achiral H_4_TPPS^2−^, and memory of the supramolecular helical chirality after helix inversion of **5**·HCl by addition of an excess amount of (*R*)-**50** in acidic water (Reprinted with permission from [117]. Copyright 2006 Wiley-VCH)

**Fig. 19:**
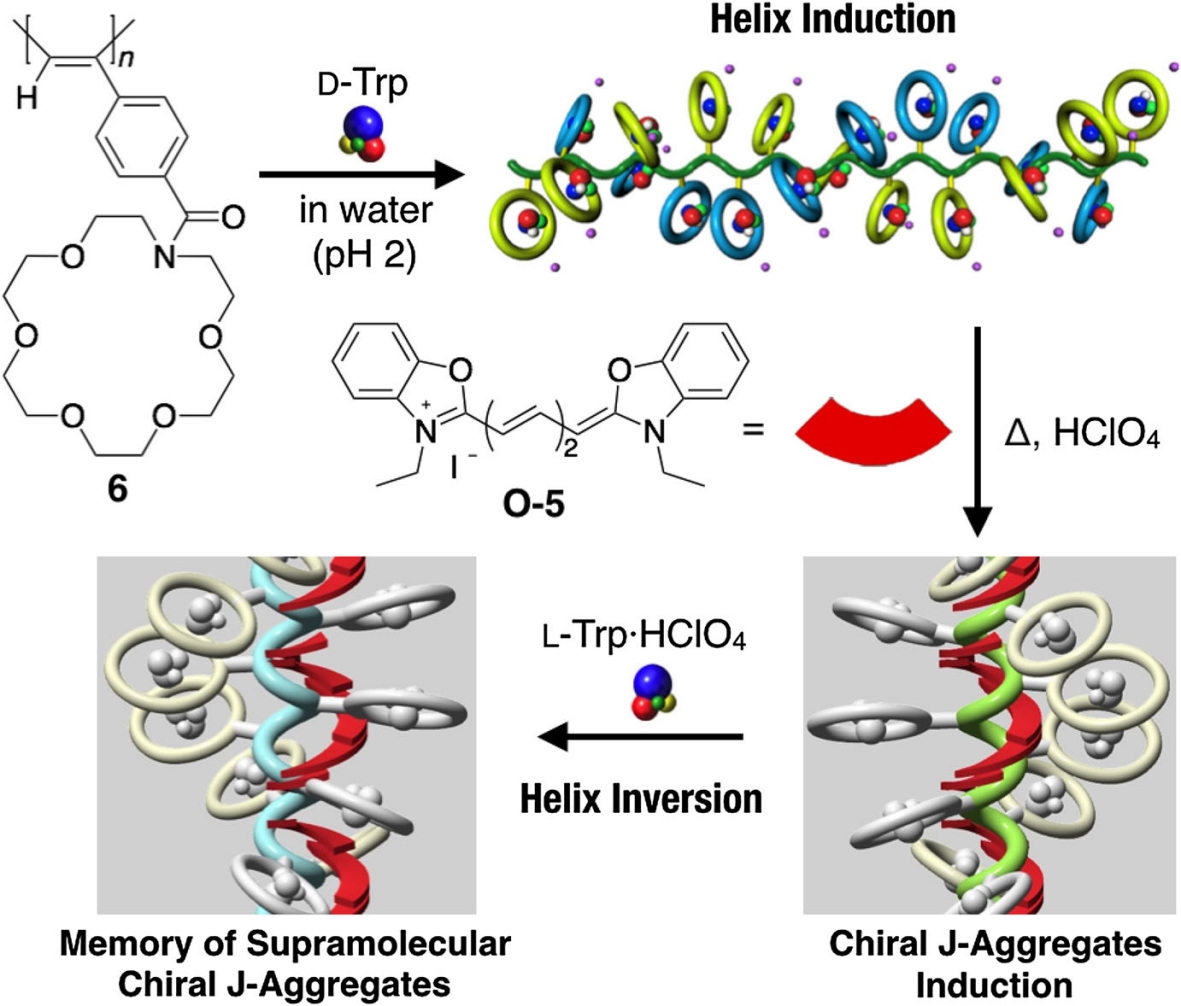
Schematic illustration of a helicity induction with an excess handedness in water-soluble **6** with D-Trp, subsequent formation of supramolecular helical J-aggregates of achiral **O-5** within the helical cavity of **6** in acidic water, and memory of the supramolecular chirality after helix inversion of **6** by addition of excess amount of l-Trp (Reprinted with permission from [118]. Copyright 2007 American Chemical Society)

By using the methodology of noncovalent macromolecular helicity induction with amplification of chirality, optically inactive polyacetylenes have been employed as a novel scaffold or template to spatially organize various functional substituents and chromophores, such as fullerene, porphyrins, and dyes, in a predominantly one-handed helical array along the polymer backbones through covalent or noncovalent bondings. The resulting unique helical hybrid materials may be applicable to optoelectronic materials as well as chiral materials for enantioseparation and asymmetric synthesis. For example, an optically inactive, C_60_-containing poly(phenylacetylene) bearing an aza-18-crown-6 ether pendant (**59**) was transformed into a predominantly one-handed helical conformation upon complexation with chiral amino acids such as l-Ala·HClO_4_, which resulted in a preferred-handed helical array of the achiral C_60_ pendants (Fig. 17a) [115]. In a complementary approach, a predominantly one-handed helical conformation could be induced in an optically inactive poly(phenylacetylene) (**2b**) with the negative charges through electrostatic interactions with an enantiomerically pure cationic C_60_-bisadduct in DMSO-water mixtures, where the bound C_60_-bisadducts are simultaneously arranged in a helical array along the polymer backbone (Fig. 17b) [116]. The water-soluble polyacetylene **5**·HCl encapsulated a hydrophobic chiral (*S*)-1,1′-bi-2-naphthol ((*S*)-**50**) within its hydrophobic cavity in water and formed a preferred-handed helical conformation. The resulting positively charged predominantly one-handed helical **5**·HCl served as a template for the further induction of supramolecular helical J-aggregates of the negatively charged achiral porphyrin (H_4_TPPS^2−^) with a predominant screw sense through electrostatic interactions (Fig. 18) [117]. The hydrophobic cavity inside the neutral water-soluble aza-18-crown-6 ether-bound poly(phenylacetylene) **6** also can be used as a template to arrange achiral cyanine dyes in a helical array. Through specific host-guest complexations in the exterior crown ether pendants, **6** folded into an excess single-handed helical conformation with d-tryptophan (Trp) in acidic water and accommodated the hydrophobic achiral cyanine dye, 3,3-diethyloxadicarbocyanine iodide (**O**-**5**), in its inner hydrophobic cavity [118]. Upon thermal annealing, the encapsulated cyanine dye formed supramolecular helical J-aggregates with a predominant screw sense along the helical backbone of the poly(phenylacetylene) (Fig. 19). Interestingly, the induced supramolecular chirality of the J-aggregates of the achiral porphyrin and cyanine dye was memorized even if the macromolecular helicity of the template **5**·HCl or **6** was inverted by the addition of excess amount of (*R*)-**50** or l-Trp, respectively (Figs. 18 and 19) [117, 118].

## Conclusion

Many helical polyacetylenes with an excess one-handedness have been synthesized based on the strategy of helicity induction through noncovalent interactions with chiral guests. They showed chiral amplification phenomena of the sergeants and soldiers effect and majority rule, in which the chiral information of the nonracemic guests can transfer with high cooperativity to induce an almost single-handed helical conformation in the polymer backbones. As evidenced by the memory effect of the induced macromolecular helicity in some polyacetylenes, the dynamic helical conformations of polyacetylenes were found to be transformed into metastable static ones because of an increase in the helix inversion barrier by introducing appropriate substituents in the pendants. It is noteworthy that the polyacetylenes showing a noncovalent helicity induction and subsequent memory effect have functioned as a helical polymer-based chiral packing material for the separation of enantiomers capable of switching their elution orders because of its switchable helicity memory effect in the solid state. This is one of the unique functionalities that may be unachievable by using conventional helical polymers possessing optically active pendants through covalent bonding. Therefore, the strategy of noncovalent helicity induction and the subsequent memory effect combined with helix inversion and/or chiral amplification phenomena that are representative characteristics of dynamic helical polymers will provide emerging opportunities for the developments of the designer helical polyacetylenes with practically useful specific functions.
